# When a viral eruption hides another one: intrafamilial outbreak of parvovirus B19 and measles virus co-infections: case report

**DOI:** 10.1186/s12879-020-05183-4

**Published:** 2020-07-11

**Authors:** Claire Grolhier, Charlotte Pronier, Arielle Belem, Julia Dina, Astrid Vabret, José-Hector Aranda Grau, Pierre Tattevin, Vincent Thibault

**Affiliations:** 1 Department of Virology, Univ Rennes, CHU Rennes, Inserm, EHESP, Irset (Institut de recherche en santé, environnement et travail), UMR_S 1085, F-35000 Rennes, France; 2 Univ Rennes, Infectious Diseases and Intensive Care Unit, CHU Rennes, F-35000 Rennes, France; 3 Department of Virology, Normandie Univ, UNICAEN, UNIROUEN, GRAM 2.0, CHU Caen, F-14 000 Caen, France; 4 Regional Health Agency – French Brittany, Direction of Public Health, F-35000 Rennes, France

**Keywords:** False positive serology, Nosocomial, Vaccine, Case report, Diagnosis

## Abstract

**Background:**

Despite high overall population vaccine coverage, identified clusters of persons refraining from vaccination interfere with pursued measles elimination. Clinical diagnosis of measles is often obvious due to its typical rash. Yet, febrile rashes may occur during many viral infections. Misdiagnosis of a specific primary viral infection may have severe consequences, particularly in immunocompromised subjects or pregnant women. To our knowledge, this case presentation is the first description of a measles and parvovirus B19 coinfection outbreak. Analysis of this outbreak underlines rash diagnosis difficulties and potential serology interpretation pitfalls. This case report is helpful for the clinicians in the context of measles re-emergence and proposes several methods to improve the diagnosis approach.

**Case presentation:**

We investigated an outbreak of rash in 6 out of 8 Traveler family members presenting to Rennes University Hospital (West of France). Anti-B19V and measles IgM/IgG antibodies were measured and detection of Parvovirus B19 and measles virus genomes were done on blood and/or respiratory samples. Virological investigations finally documented 6 cases of parvovirus B19 infections, including 4 associated with measles. Interestingly, in the four coinfection cases, the rash was typical of B19V primary infection for the two children but typical of measles for the two adults. Clinical diagnosis of rash may be misleading and thorough virological investigations may be required to avoid misdiagnosis.

**Conclusions:**

This investigation first reports an intra-familial outbreak of MeV/B19V coinfections highlighting the high transmissibility of both viruses and the diagnostic challenges of dual rash-associated infections. This report also underlines the potential deleterious consequences of failure to identify measles cases, especially in a community with low vaccination coverage.

## Background

Worldwide, unsatisfactory vaccination coverage against measles within specific communities has been associated with outbreaks, particularly in Europe. In this context, measles diagnosis is often based on initial clinical examination. Yet, febrile rashes may occur during many viral infections and maculopapular eruptions are commonly seen during parvovirus B19 (B19V), rubella virus, HHV-6, adenoviruses or arboviruses infections [1, 2]. Misdiagnosis of a specific primary viral infection may have severe consequences, particularly in immunocompromised subjects or pregnant women. In addition, failure to identify a case of measles may jeopardize implementation of adequate infection control measures, and lead to secondary transmission. In case of clinical measles suspected diagnosis, virological confirmation, relying on routine serological assays or molecular testing, is recommended to support clinical diagnosis. Indeed, it is a strong recommendation from the Global Measles and Rubella Strategic Plan 2012–2020 to enhance integrated case-based, laboratory-supported surveillance [3].

Serological testing is more accessible, but has some limitations: In the context of an acute viral infection, detection of virus-specific IgM can be impaired by IgM cross reactivity against several viruses, likely due to a lack of assay specificity. Thus, interpretation of one or several IgM positive result may be misleading between cross-reactivity and true viral coinfection. Nucleic acid amplification tests are more reliable, with much better specificity. We report an unusual intra-familial outbreak of dual B19V and measles virus (MeV) primary infections in a context of an absence of MMR (Measles, Mumps, and Rubella) vaccination.

## Case presentation

We investigated an outbreak of rash in 6 out of 8 Traveler family members, from 2 households (3 children and 3 adults). These families were settled in Brittany but had frequent interactions with families located in a neighboring French area “Pays de Loire”. They did not report any travel abroad.

Laboratory tests were performed for 6 cases. Anti-B19V and measles IgM/IgG antibodies were measured by CLIA immunoluminometry (Liaison XL DiaSorin®, Saluggia, Italy). Detection of Parvovirus B19 and measles virus genomes were done using RealStar® Parvovirus B19 PCR Kit 1.0 (Altona, Hamburg, Germany), and in-house RT-PCR (Reverse Transcription-Polymerase Chain Reaction), respectively, on blood and/or respiratory samples [4]. Three of four measles strains were sequenced by the National Reference Lab for measles in Caen Hospital.

A 34-year-old man (case 4) was admitted to the emergency department in Rennes University Hospital for a typical rash of measles, with fever. He reported that his brother (case 3) had received a clinical diagnosis of measles few days earlier. Laboratory investigations for case 4 showed anti-MeV IgM antibodies in serum, and positive MeV RT-PCR in nasopharyngeal secretions, confirming the initial clinical diagnosis. The next day, his son (case 6) came to the emergency department for rhinopharyngitis and cough associated with a febrile rash typical of primary B19V infection (slapped cheek-rash). The two diagnostic hypotheses for this child were either a B19V infection or measles. Serological testing for anti-MeV IgM antibodies returned negative but MeV RT-PCR was positive on nasopharyngeal secretions. In addition, he was reactive for anti-B19V IgM and serum B19V PCR returned positive as well. Investigating potential sources of infection, both of his parents (cases 4 and 5) were retrospectively identified as having both a primary B19V-MeV co-infection (Table 1) Retrospectively, a primary B19V-MeV co-infection was also documented by serological and molecular assays (Table 1), for both parents (cases 4 and 5).

**Table 1:**
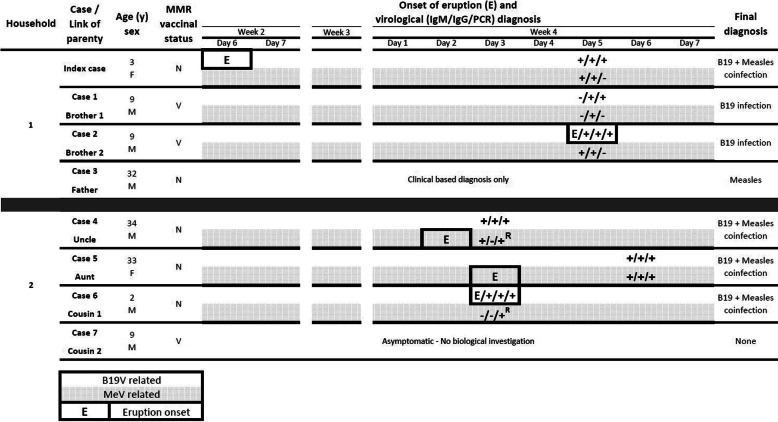
Synoptic view of the outbreak. Eruption and virological findings are indicated as black text on a white background for B19V, and as black text on a shaded background for MeV. ^R^, indicates RT-PCR results obtained on respiratory samples. Eruption onset is boxed

MMR vaccinal status: V, indicates documentation of complete MMR dose administration; N, indicates an absence of MMR vaccine administration.

Through the mandatory French Health Authorities measles case notification, we got informed that the index case for this outbreak was probably case 3’s daughter who happened to also be case 4’s niece. Initially, this 3-year-old girl had been diagnosed as a typical B19V *erythema infectiosum* by her general practitioner. Co-infection with MeV was 13 days later identified in this girl whose serum was positive for anti-MeV IgM. At that time, B19 serological tests and PCR were also performed for this girl and her twin brothers (cases 1 and 2), revealing recent primary B19V infection for both of them.

In total, 6 cases of B19V infection were diagnosed, including 4 with measles coinfection. Sequencing of three of four measles strains revealed a D8-genotype corresponding to the main circulating genotype in France by that time. Interestingly, in the four coinfection cases, the rash was typical of B19V primary infection for the two children (index case and case 6), but typical of measles for the two adults (cases 4 and 5).

## Discussion and conclusions

To the best of our knowledge, no outbreak of B19V/MeV coinfections has been reported to date. B19V and measles viruses are two highly contagious agents causing rash, with similar incubation periods. The typical rash observed during B19V infection has given rise to the name “slapped-cheek syndrome” in children [5], while measles rash classically appears first on the face and then spread to the whole body [6]. Yet, atypical presentations are not rare in clinical practice, including asymptomatic forms as illustrated by case 1 (index case’s brother). Rash description occurring during B19V/MeV coinfection is seldom reported. In these 4 co-infected cases, rash was typical of measles in co-infected adults, and typical of primary B19V infection in children. An accurate chain of transmission could not clearly be demonstrated in this investigation, as most cases were identified through passive surveillance, according to medical referral, and not by active surveillance.

Analysis of these cases highlights peculiar difficulties. While measles diagnosis was obvious for case 4 (household 2, index case’s uncle) who presented a typical eruption with confirmatory positive MeV-IgM, primary B19V infection would have been missed without additional specific serological or molecular testing. By contrast, measles diagnosis in case 6 (cousin 1) was only possible through RT-PCR testing, as anti-MeV IgM were still negative at the time of presentation. Further testing finally identified also B19V infection in this child and revealed the full extent of coinfection in this family, with 6 members presenting with virological confirmed primary B19V-infection. For the twin-brothers (cases 1 and 2), with complete vaccination against measles and infected by B19V probably at the same time, molecular tests were necessary to ascertain an accurate diagnosis. Indeed, despite negative for anti-B19V IgM and asymptomatic, the first brother (case 1) had a positive B19V-PCR. By contrast, the second brother, who presented a typical B19V eruption, had both B19V and MeV positive IgM, leading to a co-infection suspicion. Owing to the negative MeV RT-PCR at the time of eruption in a vaccinated child the conclusion was “an absence of MeV infection”.

Due to a typical eruption, initial clinical diagnosis of the index case was primary B19V infection, and no infection control measure was implemented. However, no virological testing was performed at that time, and measles was not suspected before secondary cases occurred. The 13 days delay between rash onset in the index case, and measles diagnosis, allowed the transmission of MeV to three contacts within household 2 (cases 4–6). Timely identification of index case measles could have prevented secondary cases, through respiratory isolation and early vaccination of unprotected contacts.

Although clinical diagnosis of measles is accurate when symptoms are typical, in the context of an outbreak or after a contact with a measles case, virological diagnosis is required in atypical cases or when no exposure has been reported. In addition, coinfection with two rash-associated viruses cannot be diagnosed solely on clinical grounds. The outbreak reported herein suggests that measles may be masked by a clinically prominent B19V-related rash in children, while measles-B19V coinfection in adults rather presented with typical measles rash. Identification of such co-infections is particularly relevant in the context of immunocompromised patients or during pregnancy. Indeed, the consequences of congenital parvovirus B19 infection in early pregnancy can be dramatic, as can be measles at any time during pregnancy or in severely immunocompromised patients [5].

In the context of acute infections, simultaneous detection of IgM antibodies to B19V, MeV, rubella virus, and HHV-6 has been previously reported and illustrates the sub-optimal specificity of IgM detection [7, 8]. Diagnosis of co-infection should thus rely on viral genome detection in parallel with serological tests. Multiplex PCR testing, often refered to as “syndromic testing”, may be an option for identification of rash-causing viruses and bacteria. Next-generation sequencing (NGS) could be another option, but technological and financial constraints limit this approach in clinical practice. In a recent study, NGS allowed the diagnosis of primary B19V infection among patients with a clinical diagnosis of dengue, who tested negative for all dengue virus (DENV) serology and PCR [9]. Unfortunately, most patients confirmed with DENV infection are commonly not tested by molecular assays for other rash-associated viruses, thus limiting identification of co-infections. In areas endemic for dengue, as diagnosis of dengue is mostly clinical during epidemic seasons, other viral infections associated with rash are probably missed, including transmittable diseases of public health concerns such as measles [10]. Practically, in case of a maculo-papular rash, using molecular multiplex testing on a first basis could be more efficient than a two-step process based on, first serology, followed by molecular tests. This approach will limit false positive reactions seen with serological assays and could cover most potentially involved pathogens. Yet, the choice of commercially available molecular kits is nowadays rather limited. This strategy could be extended to other viruses such as arboviruses in case of return from an endemic area and to respiratory samples whenever respiratory symptoms predominate. Obviously, any PCR-based syndromic approach should demonstrate its ability to detect most pathogens of clinical significance, with reliable sensitivity and specificity [11].

In conclusion, this first report of intra-familial outbreak of MeV/B19V coinfections highlights the high transmissibility of both viruses, the diagnostic challenges of dual infections with rash-associated viruses and the consequences of failure to identify measles cases, especially in a community with very low vaccination coverage.

## Data Availability

The datasets used and/or analysed during the current study are available from the corresponding author on reasonable request.
